# Mesenchymal to epithelial transition during tissue homeostasis and regeneration: Patching up the *Drosophila* midgut epithelium 

**DOI:** 10.1080/19336934.2016.1140709

**Published:** 2016-01-13

**Authors:** Zeus A. Antonello, Tobias Reiff, Maria Dominguez

**Affiliations:** Instituto de Neurociencias; Consejo Superior de Investigaciones Científicas (CSIC); and Universidad Miguel Hernández (UMH); Campus de Sant Joan, Apartado 18, 03550 Sant Joan, Alicante, Spain

**Keywords:** EMT, epithelial-to-mesenchymal transition, homeostasis, mesenchymal-to-epithelial transition, MET, miR-8, miR-200, progenitors, regeneration, snail, Stem cells

## Abstract

Stem cells are responsible for preserving morphology and function of adult tissues. Stem cells divide to self-renew and to generate progenitor cells to sustain cell demand from the tissue throughout the organism's life. Unlike stem cells, the progenitor cells have limited proliferation potential but have the capacity to terminally differentiate and thereby to substitute older or damaged mature cells. Recent findings indicate that adult stem cells can adapt their division kinetics dynamically to match changes in tissue demand during homeostasis and regeneration. However, cell turnover not only requires stem cell division but also needs timed differentiation of the progenitor cells, which has been much less explored. In this Extra View article, we discuss the ability of progenitor cells to actively postpone terminal differentiation in the absence of a local demand and how tissue demand activates terminal differentiation via a conserved mesenchymal-epithelial transition program revealed in our recent EMBO J paper and other published and unpublished data. The extent of the significance of these results is discussed for models of tissue dynamics during both homeostasis and regeneration.

## Introduction

The homeostatic cell turnover of adult tissues is vital to the survival of organisms.^1^ Physiological cell turnover relies on the dynamic equilibrium between the elimination of damaged or older differentiated cells and the replacement by the division progeny of adult stem cells. Deregulation of this dynamic equilibrium does temporally occur during regeneration but if sustained it can lead to tissue dysfunction (e.g. atrophy, hypertrophy or cancer).^2-5^ Stem cells are responsible for maintaining this balance throughout organisms' life by perpetuating the cell lineage, number and type of new cells. The capacity to maintain stem cell lineages is achieved through self-renewal and requires preservation of an undifferentiated state.^6,7^ The ability to replace different cell types is dependent on the capacity to terminally differentiate of the committed progeny (named progenitor or precursor cells according to the model system and grade of differentiation). Progenitors often share similar cellular properties and markers with stem cells, however they lose long-term self-renewal capacity and progressively terminally differentiate.

Self-renewal and cell differentiation are the 2 fundamental processes sustaining the homeostatic turnover or regeneration of cells that die due to wear and tear or acute damage, respectively. During homeostatic turnover, cells loss are replaced without any evident structural and functional changes.^8^ During regeneration, tissue structure and function are temporarily lost and subsequently restored albeit often imperfectly. ^9^ In this situation, feedback mechanisms from the tissue signaling to stem cells ensure faster proliferation rate to meet the increased tissue demand. Although it is generally assumed that similar feedback mechanisms might sustain homeostatic turnover, no visible changes of such signals have been detected and as such it remains unclear to which extent these regeneration and homeostatic tissue maintenance resemble each other.^8^ Specifically, during homeostatic turnover, it is unclear whether progenitors differentiate steadily upon stem cell division as in regenerative paradigms. And if not, what mechanisms control the timing of progenitor's transition from the stem-like cell state to the post-mitotic-terminally differentiating cell.

Likely due to technical challenges to monitor the dynamics of native stem cells in steady state, most models of tissue homeostasis are based on regenerative data or indirect functional assays such as transplantation. Stem cells proliferation and differentiation are strongly coupled events in regenerative paradigms.^10-12^ Therefore, these models take a stem cell-centric perspective, placing high importance on the feedback signals modulating stem cell proliferation rate, mode of division (symmetric versus asymmetric) and lineage commitment,^13-17^ while it still remains unclear how the process of terminal differentiation and replacement of the cell loss occurs and is modulated to ensure proper homeostatic cell turnover.

*Drosophila* posterior midgut is emerging as a useful model to investigate homeostatic cell renewal.^10^ First, the existence of intestinal stem cells (ISCs) has recently been established in the adult *Drosophila*, an organism suitable for profound genetic analysis and manipulation. Second, the identification that only ISCs but not their committed progeny divide has simplified the analysis of mechanisms and pathways regulating stem cell division. Third, genetic and molecular analysis of the *Drosophila* intestinal repair and regeneration after injury, stress, or infection provide evidence that fly intestinal homeostatic and regeneration rely on conserved signaling pathways and factors originally described in stem cells and progeny of other *Drosophila* or mammalian tissues.^10^ These studies show that after division of ISC, the 2 daughter cells adopt the stem cell or the committed progenitor cell fate (also called Enteroblasts, EB). In the *Drosophila* midgut, enteroblast does not divide and can terminal differentiate into an absorptive enterocyte or a secretory enteroendocrine cell, through incompletely understood mechanisms.

Insights into the mechanisms and factors underlying physiological cell turnover depend on tools and techniques for lineage tracing of native stem cells. Recently, we have reported a novel tracing method in *Drosophila* virtually applicable to any other stem cell system, named ReDDM (Repressible Dual Differential stability cell Marker).^18^ This method provides accurate monitoring of the stem cell population and simultaneous observation of cell turnover events, both in homeostatic and regenerative conditions. Our results highlight the importance of spatiotemporal control of progenitor cell differentiation, which needs to be integrated in the actual paradigms and models of tissue homeostasis and regeneration.

## ReDDM Highlights Spatiotemporal Flexibility in Progenitor Cell Terminal Differentiation

The ReDDM lineage tracing approach is unique in the manner of labeling stem cells and their progeny in a genetic and hierarchical manner. The use of fluorescent proteins of different color, protein stability, and cellular localization, allows easy visualization of both undifferentiated cells and newly differentiated progeny simultaneously but in a mutually exclusive manner: double labeling with membrane-tethered GFP and nuclear H2B-RFP is restricted to stem and progenitor cells while nuclear H2B-RFP is retained in differentiated progeny. The approach allows highlighting stem and progenitor cells morphology and permits examination of tissue turnover with single cell resolution with automated systems of image analysis. ReDDM can be combined with stem cell and/or progenitor cell Gal4 driver lines and also coupled with UAS-transgenes to overexpress or UAS-RNA interference lines to downregulate candidate genes specifically in the Gal4-driven stem/progenitor cells while allowing tracking the differentiation of daughter cells without directly altering them genetically. Importantly, while the MARCM technique, which is commonly used strategy to follow stem cells behavior in the midgut, labels only a subpopulation of dividing stem cells at the moment of clonal induction, the ReDDM approach labels the entire stem cell and progenitor population and their ‘production’. This not only results in a high and precise number of observed cells, but also enables the experimenter to acquire spatial data and to reveal eventual local variations in differentiation rates within the investigated epithelium. In summary, the ReDDM approach is a platform that provides a comprehensive picture of the whole stem cell and progenitor population during *Drosophila* midgut epithelium replenishment.

Using ReDDM in age synchronized co-cultured fruit flies we showed that the distribution, size and shape of the renewed areas were not homogeneous, varying greatly from intestine to intestine. These variations were noticeable when comparing the same area of the midgut (i.e. the posterior midgut) therefore excluding a behavior linked to a different regional identity as illustrated in the case of the stem cells of the copper cell region (CCR).^19^ This variation likely reflects that chance plays a key role in which intestinal cell dies and which cell survives and that intestinal renewal is not hardwired pattern and is not predictable. In fact, we observed that progenitor cells were abundant also in areas where replenishment was not occurring, suggesting that they could defer their differentiation in the absence of local demand. These observations revealed an unsuspected plasticity of progenitor cells, which could differentiate or postpone differentiation according to local demand. Indeed, using Flyblow multicolor labeling method combined with the MARCM clonal analysis we could label individual stem cell-derived clones and progenitor single-cell clones to comprehensively determining that progenitor cells do not differentiate according to their birth time but in reaction to a local demand. Thus, Flyblow clones enabled us to detect single-cell clones, which represent labeling of progenitor cells, which have retained an undifferentiated state for up to 2 weeks. These cells exhibited increased ploidy, suggesting they were in the process of differentiating into an absorptive enterocyte, but also retained characteristic of undifferentiated cell morphology and were not intercalated within the epithelium. Additionally, Flyblow clones confirmed the non-homogenous patterns of intestinal replenishment highlighted by the ReDDM method. Furthermore, we could visualize the morphologies of individual stem cell-driven renewed area and to track the differentiated lineages. Although most clones retained spatial contiguity in accordance with a ‘local’ replenishment by a individual stem cells and their progenitor cells, we also observed occasional intermingling of cells from neighboring clones and fragmentation of some clones, indicating that progenitor cell migration and positional re-adjustment are part of the replenishment process. Together, ReDDM analysis and Flyblow clones indicate that terminal differentiation and replacement are individual decision-making processes.

## Epithelial Homeostasis and Regeneration Require Mesenchymal-To-Epithelial Transition of Progenitors

**Figure 1.**
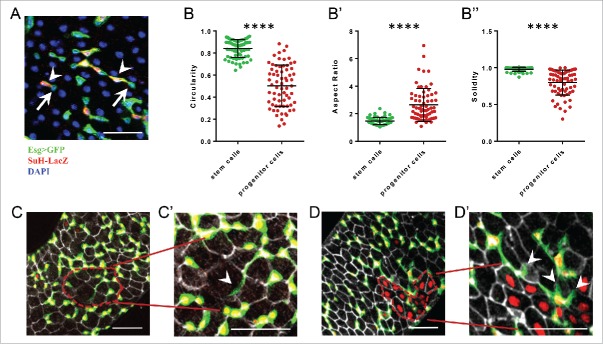
Progenitor cells have a marked planar cell polarity characterized by long protrusions and can sense the surrounding epithelial cells determining where to differentiate. A) Intestinal stem (ISC) and progenitor cells (EB) are marked by escargot-GAL4>UAS-CD8::GFP while EB co-stain with a Su(H)-LacZ reporter. Arrows indicate ISCs, arrowheads EBs. B-B″) Comparative morphological analysis of stem vs. progenitor cells. Bars represent mean and standard deviation of the mean. B) Circularity describes how close the relation between the area and perimeter of the cell shape is to that of a perfect circle. B′) Aspect ratio reflects the degree of elongation. B″) Solidity describes convexity of the cell shape. C-C′) An EB sending a long protrusion (arrowhead) toward a not yet replenished area which has lower density of EBs (red dashed line). D-D′) EBs which protrusions are likely repulsed (arrowheads) from areas which have already been replenished (red dashed area). **** = p-value <0.0001 (2 tails unpaired T-test). Scalebar = 50 µM.

The *escargot*-ReDDM marking of stem and progenitor cells takes advantage of a membrane tethered GFP originally designed to highlight neuronal processes.^20,21^ This approach facilitated the comprehensive analysis of the cellular architectures of intestinal stem cells (ISCs) and committed progenitor cells (enteroblasts, EB). As previously described, ISCs are small round cells that are basally located in the midgut epithelium.^22-24^ However, in stark contrast, progenitor cells have a front-rear polarity and extend long protrusions and branching, typical of migrating mesenchymal cells (**Fig**. **1**). In addition, using *ex-vivo* live imaging of whole intestine, we detected occasional movements and repositioning of progenitor cells, further highlighting not only the mesenchymal traits but also the mesenchymal behavior of progenitors. Finally, terminal differentiation involved a mesenchymal-epithelial transition and the repression of the mesenchymal factors escargot and zfh1 and the activation of epithelial markers such as Dlg-1 (Antonello et al. 2015).

**Figure 2.**
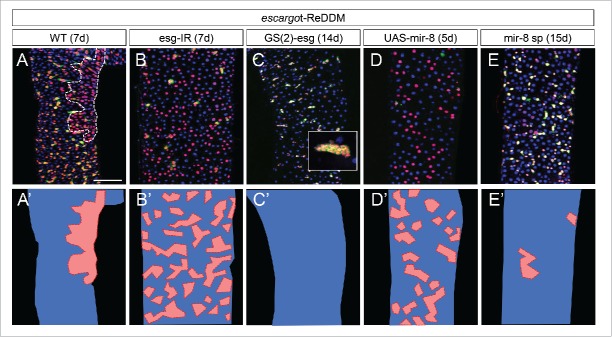
Genetic manipulation of the escargot-miR-8 balance alters the spatial and timing control of progenitor's differentiation. A-E) Esg-ReDDM analysis of escargot and miR-8 loss and gain of function conditions compared to wild type controls at the indicated time points. A'-E') cartoons summarizing the tissue replenishment phenotype of these genetic conditions. Red represent a renewed ‘patch’ of tissue, blue non-renewed parts. A-A') Tissue replenishment in controls occurs by patches (dashed red line outlines a discrete patche of new ECs) while downregulation of Esg (B-B') or overexpression of miR-8 (D-D') lead to increased and homogeneous replenishment pattern. Gain of function of Esg (C-C') leads to total block of replenishment leading to tumors in 40% of flies (as show in the inset panel). Loss of function of miR-8 (E-E') by the miR-8 sponge (mir-8sp) construct overexpression leads to a comparable phenotype to the Esg gain of function, but no tumor formation within 2 weeks of ReDDM tracing. Scale bar = 100uM.

Escargot is an ancient member of the snail family of transcriptional repressors,^25,26^ that are master regulators of the epithelial to mesenchymal transition (EMT) during development and disease.^27-29^ In human and murine cell cultures, pro-epithelial microRNAs of the miR-200 family have been shown to be down-regulated in cancer stem cells and normal epithelial cells.^30^ In addition, the miR-200 family and the EMT inducer ZEB1 have been reported to reciprocally repress each other and control the motility an invasive behavior of cancer cells.^31-33^ *zfh-1* and *mir-8* are fly homologues of the mammalian ZEB1,2 and miR-200 family, respectively.^34,35^ *escargot*, together with *zfh1*, are crucial players in midgut homeostasis controlling both stemness and the undifferentiated state of progenitor cells. The microRNA mir-8 triggers terminal differentiation in response to tissue demand by directly repressing the mesenchymal factors *escargot* and *zfh1*. As such, when *escargot* or *zfh-1* are downregulated or the microRNA *mir-*8 is activated in ISCs and EBs, stemness was rapidly lost (as also reported for *escargot* by Korzelius J. et al. ^36^ and Loza-Coll at al. ^37^) and progenitor cells lost their mesenchymal traits and acquired epithelial state. Importantly, unlike the patchy and erratic pattern of cell renewal seen in normal homeostasis (**Fig. 2A**), upon genetically suppressing mesenchymal traits, the newly differentiated cells followed a homogenous salt and pepper distribution (**Figs. 2B and D**), likely reflecting the in situ transformation of ISCs and EBs into differentiated cells. When *escargot* (**Fig. 2C**) or *zfh-1* (see suppl. Information in Antonello at al. ^18^) was overexpressed in ISCs and EBs or the microRNA *mir-8* was suppressed (**Fig. 2D**), all ISCs and EBs retained stemness and strongly reduced terminal differentiation, blocking cell turnover.

We also presented evidence that mechanical forces are sufficient to drive activation of expression of microRNA mir-8 in pre-existing progenitor cells, resulting in enhanced epithelial cell renewal. This may provide a starting point for future research in intestine epithelial cell replacement. In particular, according to the ‘apoptotic force theory’,^38^ mechanical forces and not only chemical signals, may coordinate epithelial cell loss with terminal differentiation. As we proposed, progenitor cells extend cellular protrusions that make contact with epithelial cells. A progenitor cell via cell-cell contact mechanisms may be able to sense the damage of its neighboring mature cell, in this manner ensuring that only one progenitor responses and integrates to fill in the space left by the dying cell. The forces contributing to the clearance of the dying cell and the sequential cell replacement event, may persist until homeostatic tension has been restored, explaining the occurrence of patches of newly renewed cells rather than isolated cell renewal events. This framework provides a conceptual shift of the current stem cell-centered view of tissue renewal ensuring the balance between epithelial cell loss and addition of new cells.

Overall, our data indicates that the morphological and motile properties of progenitor cells, together with the postponement of differentiation, are required for tissue replenishment and are mechanistically linked to the mesenchymal to epithelial transition program. In detail, our data indicated that both during homeostasis and regeneration (1) *escargot* and *zfh-1* are sufficient and required to retain undifferentiated state of stem cells and progenitors; (2) *mir-8* is sufficient and required to repress undifferentiated state and to promote transition toward epithelial state; and, (3) mesenchymal traits are required to maintain progenitor cells plastic to timely and spatially respond to localized tissue demand.

## Conclusions and Implications on Models of Tissue Dynamics and Regenerative Medicine

We found that in normal homeostatic conditions, *Drosophila* physiological midgut cell turnover follows inhomogeneous, unpredictable pattern reflecting the stochastic nature of events damaging mature cells. Importantly, progenitor cells seem to be the key targets of tissue demand and we have shown that progenitor cells have mesenchymal features and extend dynamic cellular protrusions to monitor their environment. Progenitor cells have also the ability to postpone their terminal differentiation and to migrate short distances to the site of cell loss. All these properties rely on a mesenchymal-epithelial program involving the Escargot/Snail2, Zfh-1/ZEB and the miR-8/miR-200 microRNA. Escargot enables progenitor cells to hold the undifferentiated state, also repressing the *mir-8* locus, and confers progenitor cells mesenchymal traits as well, which we found to be a prerequisite for proper intercalation into the epithelium. Conversely, miR-8 can directly repress *escargot* mRNA leading to the transition from the undifferentiated to differentiated (epithelial) state by suppressing the mesenchymal characteristics. Disruption of the Escargot-miR-8 regulation impacts on intestinal homeostasis by altering the spatial and timing control of progenitor's differentiation (**Fig. 2**) and affecting organism's survival.

A main shift in the paradigm is that committed progenitor cells are active players in homeostasis and not solely transient and passive entities. The mesenchymal phenotype of enteroblasts indeed allow dynamic monitor of their surroundings. For example, the enteroblasts cellular protrusions make cell-cell contact with the epithelial cells and could detect changes in tension and mechanical forces generated during the elimination of a dying cell.^38,39^ Mechanical inputs would lead to the activation of expression of genes involved in-terminal differentiation such as the microRNA *mir-8* in the progenitor cell that make contact with the dying cells. This will ensure the promotion of terminal differentiation and intercalation into the epithelium to precisely fill the ‘gap’ of the cell that died.

In summary, our findings suggest that (1) progenitor's differentiation does not occur passively or is determined by the time of birth, but rather it is activated in response or reaction to a cell loss (differentiation ‘on demand’); (2) the progenitor's mesenchymal feature enables the progenitor cells to sense ‘on demand’ signals. Interestingly, stem cell proliferation and terminal progenitor cell differentiation can also be driven by systemic hormones such as the juvenile hormone in response to mating to remodel the size of the posterior midgut.^40^ Future research will focus on deepening our knowledge on the importance of classical niche and feedback signaling-, hormonal- and mechanical pathways controlling midgut homeostasis. Conceivably, progenitors may be able to integrate local feedback signals to regulate their cellular state through intrinsic transcriptional factors and regulatory microRNAs.

Finally, given the conservation of the escargot/zfh1/miR-8 axis in mammals, we believe that insights into the identity and cellular mechanisms controlling escargot and miR-8 activation and downstream genetic program will be relevant for future understanding of tissue homeostasis and the physiological and pathological mesenchymal to epithelial transition process during wound healing and cancer.
